# Digital Platforms in the Assessment and Monitoring of Patients with Bipolar Disorder

**DOI:** 10.3390/brainsci7110150

**Published:** 2017-11-12

**Authors:** Arvind Rajagopalan, Pooja Shah, Melvyn W. Zhang, Roger C. Ho

**Affiliations:** 1 Imperial College School of Medicine, South Kensington Campus, London SW7 2AZ, UK; arvind.rajagopalan11@imperial.ac.uk (A.R.); Pooja.shah13@imperial.ac.uk (P.S.); 2 Biomedical Global Institute of Healthcare Research & Technology (BIGHEART), National University of Singapore 119228, Singapore, Singapore; melvynzhangweibin@gmail.com; 3 Department of Psychological Medicine, Yong Loo Lin School of Medicine, National University of Singapore, Level 9, NUHS Tower Block, 1E Kent Ridge Road, Singapore 119228, Singapore

**Keywords:** digital platform, smartphone, application, mobile platform, mobile application, mobile app, smartphone application, smartphone app, bipolar disorder, bipolar disorder app, psychiatry app, mental health app

## Abstract

This paper aims to review the application of digital platforms in the assessment and monitoring of patients with Bipolar Disorder (BPD). We will detail the current clinical criteria for the diagnosis of BPD and the tools available for patient assessment in the clinic setting. We will go on to highlight the difficulties in the assessment and monitoring of BPD patients in the clinical context. Finally, we will elaborate upon the impact that diital platforms have made, and have the potential to make, on healthcare, mental health, and specifically the management of BPD, before going on to evaluate the benefits and drawbacks of the use of such technology.

## 1. Introduction

Bipolar disorder (BPD) is a psychiatric illness with a significant global burden, with an incidence rate of 2.6% in United States [1,2]. Globally, the disorder is reported to have an aggregate lifetime prevalence of 2.4% [3]. The disease burden is slightly lower in Singapore, with a reported prevalence of 1.2%. The high global prevalence of BPD is associated with significant costs. The total economic burdens of the disorder in the US and UK in 1991 were estimated at USD 45 billion and GBP 2 billion, respectively [4,5]. The aetiology of BPD is still unclear but various biochemical, pharmacological, anatomical, and genetic theories have been postulated [6]. There is a clear genetic association as the concordance rate in monozygotic twins is 70% compared to 20% in dizygotic twins [7]. The largest twin study reported a heritability estimate of 85% [8]. Other theories include neurochemical abnormalities (hypothalamic–pituitary–adrenal axis dysfunction), structural brain differences, and psychosocial influences [7]. The most consistent structural findings are: preservation of total cerebral volume with regional grey and white matter changes in prefrontal, midline, and limbic networks; non-contingent ventriculomegaly; and increased rates of white matter hyper-intensities [9]. While advances in drug treatment for this disorder remain modest at best, substantial progress has been made in the development of adjunctive psychosocial interventions [10]. This paper will review the diagnosis, assessment, and monitoring of BPD and the use of digital platforms in these endeavours.

### Methodology

This is a status quo review. The intention of this paper is to present the most current research on the topic of digital platforms and their use in the diagnosis and management of BPD. Electronic Databases-PubMed, Ovid EMBASE, and Cochrane Library were searched for the terms “Bipolar disorder and smartphone”, “Bipolar disorder and digital”, and Bipolar disorder and internet”. 

The studies were reviewed by one of the authors for relevance and adherence to the inclusion criteria:

1. Randomised controlled trial or observational study
2. Authors explicitly suggest use of a digital platform
3. The study controlled for at least 1 confounding factor e.g., age, socio-economic status
4. Study published between 01 January 2005 and 01 September 2017
5. English language journal.

**Figure 1:**
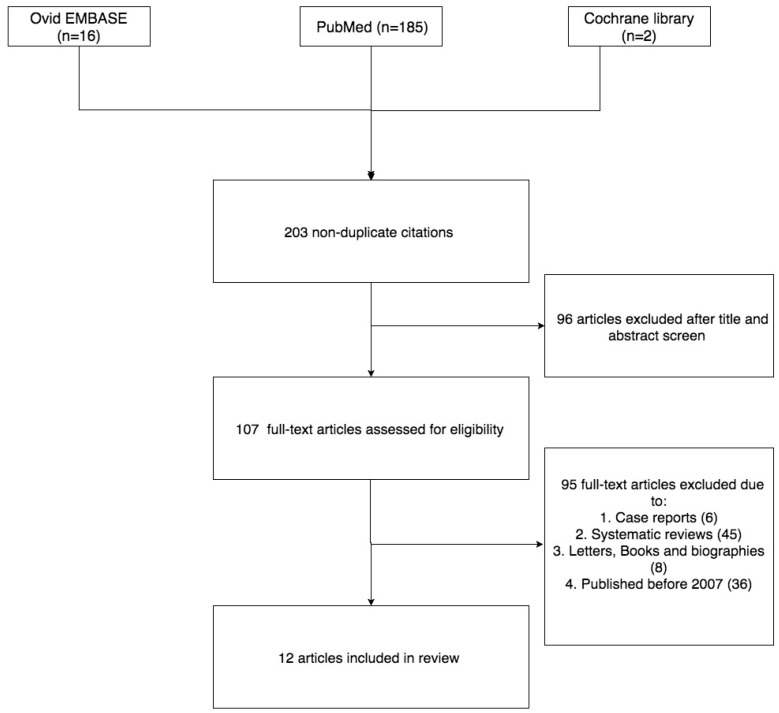
Figure explaining the selection of studies included in the review.

The searches produced 200 citations, of which 137 were considered relevant. The remaining articles were discarded, as they simply discussed general use of web-based platforms without mention of a specific intervention. Out of the 137 articles, 15 studies met the inclusion criteria (Figure 1). Study characteristics are described in Table 1. 

## 2. Diagnostic Criteria of BPD

Currently, there are two guidelines used in the diagnosis of BPD: the tenth revision of the International Statistical Classification of Diseases and Related Health Problems (ICD-10), and the Diagnostic and Statistical Manual of Mental Disorders, Fifth Edition (DSM-V). These are used by the World Health Organisation and American Psychiatrists’ Association, respectively, and both classify BPD into subsets.

### 2.1. ICD-10 Criteria

BPD, specified as Bipolar Affective Disorder, is designated as F31, with numerous subtypes (F31.0–F31.9) [23]. The diagnostic criteria include:

- At least one episode must be characterised by elevated mood and energy/activity levels (hypomania/mania)
- Other episodes can be depressive (low mood, low energy/activity levels) or manic/hypomanic in nature
- Two or more episodes of significant disturbance of mood and activity levels.

### 2.2. DSM-V Criteria

The DSM-V designates BPD with the number 296, under which there are four major sub-classifications: Bipolar I Disorder, Bipolar II Disorder, Cyclothymia, and Bipolar Disorder Not Otherwise Specified (BPD NOS) [24]. Table 2 below illustrates the diagnostic criteria for the different sub-classifications of BPD:

As such, current diagnosis of BPD is based purely on clinical presentation, including symptomatology and functional capacity. Diagnosis therefore requires observation of patients by clinicians regularly in in-patient or out-patient settings.

## 3. Assessment and Monitoring of BPD

### 3.1. Need for Early Diagnosis

BPD is associated with significant psychological and social morbidity. Patients with the disorder are known to experience more difficulties with social or family interactions, leisure activities, and work life. Men with BPD are more likely to be convicted of crimes and arrested [25]. These patients are also less likely to report improvements and have lower responses to treatment, making clinical follow-up challenging [26,27]. These patients also suffer from numerous psychological comorbidities including anxiety, substance abuse, and personality disorders [28]. Tellingly, delayed treatment (a consequence of delayed diagnosis), may result in patients never recovering full social functioning [25,28]. BPD is also associated with increased mortality, as patients are more likely to exhibit suicidal behaviour [29]. 

Thus, the importance of early diagnosis of BPD is clear. Regardless, many cases of late diagnosis, and subsequently delayed treatment, are still reported [30]. There are two main reasons for this: Firstly, patients are unaware of the characteristics of the disorder, and therefore delay seeking medical attention [31]. Furthermore, patients may also miss clinic appointments, preventing subsequent follow up. Secondly, at first presentation, BPD is often misdiagnosed, the reasons for which are detailed in the next section [32]. In such cases, treatment can be delayed for as long as ten years from the onset of symptoms [32,33].

### 3.2. Screening Tools

There are a few points in the course of BPD at which early detection may be possible, between the development of prodromal features until the time symptoms match diagnostic criteria. The difficulty with identifying the prodromal stage lies in the non-specificity of presenting symptoms; many symptoms of early BPD can also be seen in normal reactions to stressful situations and are hence not necessarily diagnostic. Furthermore, these symptoms are also seen in other psychiatric presentations. Patients in the prodromal stage can also fluctuate and can hence present as completely normal, depending on when they are seen by clinicians. The low specificity and fluctuating course of prodromal symptoms would lead to more false positive diagnoses as well as drop-outs from care [30]. Numerous studies are investigating the role of specific genes and biomarkers in the development of BPD, but these are still some time away from being diagnostically useful [34,35].

As such, current screening tools are focussed purely on clinical presentation and patient experience of the illness, taking the form of questionnaires and scoring scales to judge the severity of the disorder in individual patients.

#### 3.2.1. The Mood Disorder Questionnaire (MDQ)

The MDQ is a self-reporting form for patients that has been developed based on DSM-IV (fourth edition) criteria for BPD as well as clinical expertise, which has been shown to have both good sensitivity and specificity for BPD. The MDQ form consists of thirteen “yes/no” questions in addition to items assessing functional impairment and the clustering of symptoms. Positive responses to questions addressing symptom clustering, along with seven of the thirteen core question, provides high specificity and sensitivity for BPD [36].

#### 3.2.2. The Bipolar Spectrum Diagnostic Scale (BSDS)

Unlike the MDQ, the BSDS is based on scenarios or narratives that are descriptive of specific mood states. Patients are asked to identify the narrative that describes their mood from nineteen different statements describing different states. Based on their responses, they are given a score, out of nineteen, which predicts the likelihood of them having BPD. The lower the score, the higher the probability of BPD [36]. While the MDQ has been shown to be more sensitive to Bipolar I than Bipolar II disorder, the BSDS is equally adept at identifying both, which may be attributed to the narrative-based approach being more useful in distinguishing subtler hypomanic symptoms [37].

#### 3.2.3. The Hypomanic Personality Scale (HPS)

The HPS is another self-reporting questionnaire for patients, consisting of forty-eight “true/false” questions [36]. It has been shown to be a good predictor of BPD and hence may have more value as a screening tool in the psychiatric setting, seeing as it only screens for hypomanic symptoms [38]. However, the length of time taken to complete all of the forty-eight questions is an impediment to its usefulness in this setting [36].

### 3.3. Monitoring Tools

There are considerable hurdles in developing formalised monitoring systems for BPD. Formal rating scales are unsuitable for use in routine clinical monitoring, as they tend to focus on limited and specific aspects of BPD, are time-consuming, and cannot provide a prospective view. Therefore, they are unable to capture much of what is needed for monitoring. Furthermore, patients may experience different features of BPD over a lifetime, such as rapid cycling, mixed episodes, and psychoses. This makes the development of a generalised assessment tool for individual patients at different points in the course of the disorder difficult [36]. There are two monitoring tools currently available, both of which were initially developed for use in research: The National Institute of Mental Health Life Chart Methodology (NIMH-LCM) and the Clinical Monitoring Form (CMF). 

#### 3.3.1. The National Institute of Mental Health Life Chart Methodology (NIMH-LCM)

The NIMH-LCM is a tool that allows for daily assessment of mood severity based on mood-associated functional impairment [39]. By providing both a prospective and retrospective take on the course of the illness in individual patients, this tool allows for both the identification of risk factors which may trigger severe episodes as well as assessment of the efficacy of medication. However, patients require adequate training and guidance to use this tool, and the large amount of data entry required may result in it being too tedious and expensive for routine use [36]. As such, an electronic version of the NIMH-LCM has been developed, and recent studies have found that patient self-assessment using the tool is a valid, cost-effective alternative to clinician-use versions [40,41]. 

#### 3.3.2. The Clinical Monitoring Form (CMF)

The CMF is an eight-part tool with specific sections including mood status, comorbidities, and medication use, allowing for a comprehensive evaluation of patients with BPD [36,42]. It has been used in large, multicentre trials on BPD management, such as the Systematic Treatment Enhancement Programme for Bipolar Disorders (STEP-BD) trial [43]. As with the NIMH-LCM, electronic versions of the CMF are being developed [36].

In general, the difficulty with the abovementioned assessment and monitoring tools in a clinic setting is that firstly, the rely greatly on subjective data, such as the patients’ accounts of their moods and energy levels, and secondly, the objective data they attempt to obtain, such as activity levels and sleep patterns, is dependent on retrospective accounts by patients, which reduces the objectivity of said data. 

## 4. Digital Platforms in Medicine

It is estimated that there are more than three phones for every person in the world on the market [44]. The ubiquitous nature of smart mobile devices and applications has allowed for the usage of these platforms in the improvement of healthcare and the medical practice [45]. In 2014, companies operating in the digital healthcare space generated over four billion dollars in venture funding, approximately as much as in the previous three years combined, indicating that digital healthcare is a field that is gaining commercial interest. However, the question remains as to whether commercialisation of digital health applications would lead to truly disease-based interventions, or simply consumer-based products [45]. In general, digital platforms have three main uses in healthcare: digital phenotyping, remote monitoring, and remote management.

### 4.1. Digital Phenotyping

In healthcare, phenotyping involves the use of known biological markers of diseases to assess patient health. Examples include using blood glucose levels to assess the severity of diabetes. Digital platforms have allowed for the gathering of new types of physiological data not previously accessible. This new information, coupled with existing knowledge, could prove to be useful in the detection of disease [45]. For example, research has shown that high amyloid burden at baseline in Alzheimer’s patients correlated with a three-year cognitive decline, as measured by repeated computerized cognitive tests. This suggests that such digital cognitive tests can act as neural correlates for the monitoring of disease progression in Alzheimer’s disease [46]. Companies such as Pfizer and Akili Interactive Labs are combining traditional phenotypic biomarkers obtained through neural imaging, with digital phenotyping through video game measurements, to obtain a prodromal profile for Alzheimer’s disease [45,47]. 

### 4.2. Remote Monitoring

Wearable technologies and digital platforms have allowed for continuous real-time monitoring of patients’ symptoms. This immediately allows for advantages over traditional periodic monitoring by physicians. Firstly, symptoms can be monitored on a more regular basis, with built-in alarms to detect increasing severity, allowing for earlier and more rapid response. Secondly, this form of monitoring can be coupled with mobile applications designed as preventative tools. Finally, from a research perspective, continuous monitoring allows for faster collection of more data, which may reveal new patterns of symptomatic or biomarker changes that are indicative of disease progression. There are already numerous mobile applications on the market which allow for physiological monitoring through sensors built into the mobile platforms, such as AliveCor’s^©^ electrocardiogram, Kinsa’s Smart Thermometer^©^, and Sanofi’s iBGStar^©^ glucose monitor [47]. Wireless wrist monitors that monitor blood pressure that sync directly to smartphone applications are now available, and wearable defibrillators for post-myocardial infarction patients are under development [48,49]. 

### 4.3. Remote Management

As mentioned above, continuous monitoring can help with earlier intervention in patients. Real-time data can allow for monitoring of not just disease processes, but also risk of disease, and therefore result in intervention that is preventative rather than reactionary. Primary prevention is usually based on behavioural modification, with a few exceptions such as prophylactic antibiotics and statins to prevent infection and hypercholesterolemia, respectively [45,50,51]. Behavioural modification is an intervention that can potentially be assisted by digital platforms. For example, Propeller Health has developed a remote wireless sensor attached to bronchodilator inhalers that delivers real-time data on timing and locations of usage in patients with asthma or chronic obstructive pulmonary disease [52]. This technology then generates reports for patients on a regular basis and suggests behavioural modifications based on guidelines, which has been shown to significantly reduce inhaler usage and improve disease control [52,53]. As mentioned above, Akili Interactive Labs is using video game platforms to digitally monitor cognitive function in patients with Alzheimer’s disease [46]. Their research indicates that multitasking abilities, assessed by their custom-designed video game NeuroRacer^©^, shows age-related decline from the age of twenty to seventy-nine. They have since found that training these abilities using an adaptive version of NeuroRacer^©^ improved multitasking abilities and also improved electroencephalographic markers of cognitive function, sustained attention, and working memory [47]. It is possible that such technologies can not only allow for earlier detection of cognitive decline, but also for non-pharmacological management of this decline, both of which are major challenges in the management of Alzheimer’s disease. The development of gaming-based interventions, such as NeuroRacer^©^, can also be done in a way that is integrative with existing gaming engines, making it more cost-effective as well [54].

### 4.4. Digital Platforms in Psychiatry

A recent paper has found that the psychiatric profession has embraced the use of digital platform technologies in the management of psychiatric conditions. It proposes that the field of psychiatry not only has a good scope for the use of technologies such has smartphone applications, but that psychiatrists can actually get involved directly in the development of these applications [55]. In fact, there has been research published by clinical psychiatrists on possible methodologies for psychiatric application development for mobile phones [54]. Applications for many psychiatric presentations, both acute and chronic, have already been developed and trialled. A trial of forty-four patients with schizophrenia found that the use of a smartphone application to allow patients to self-report psychotic symptoms was a feasible means of ambulatory monitoring of these patients [56]. Psychiatrists have also proposed models for smartphone applications to assist in the community management of patients with dementia [57]. Similarly, models for applications have also been proposed to assist with addiction issues. For example, an alcohol tracker application has been conceptualised, which functions to warn users when their blood alcohol levels are approaching recommended limits [58]. Similarly, eGambling Intervention, a smartphone application for compulsive gamblers, has been conceptualised by a team in Singapore. It makes use of the in-built Global Positioning Satellite (GPS) system in the mobile phones, notifying users when they are in proximity to gambling dens in the country [59]. Applications to assist in the management of internet addiction are currently being conceptualised as well [54]. Mobile phones are also being used as platforms for screening to be carried out using existing screening tools. For example, a depression screening tool that contains the patient health questionnaire PHQ-9 has been developed and validated [60]. Finally, smartphone applications are also under development for acute psychiatric presentations, such as delirium [61].

## 5. Digital Platforms for BPD

From above, it is clear that digital platforms provide a number of advantages:

- Risk assessment for earlier diagnosis of new cases and monitoring of known cases
- Discovering new markers of disease progression to allow for more effective monitoring
- Remote management with early or preventative intervention via targeted behavioural modification.

As mentioned previously, the main difficulties in the management of BPD are late diagnosis, primarily due to non-specificity of symptoms in the early stages and misdiagnosis, and difficulty in monitoring response to treatment, due to the fluctuating nature of the disorder. A study assessing community attitudes towards the use of mobile phone applications for mental health disorders found that the majority of participants (76%) were interested in using these for self-monitoring and management of their conditions [62]. More recently, a study of 89 eighteen- to thirty-year-old adults with BPD found that 79% of those not using smartphone applications for disorder management wanted to try them, and that 61% of the self-management strategies provided by these applications were listed as desirable by these participants [63]. 

### 5.1. Self-Monitoring and Risk Assessment

#### 5.1.1. Monitoring Patients with Known BPD

A review of 82 mobile applications for BPD found that more than half of these were designed specifically for symptom monitoring, screening, and assessment [64]. A simple text message-based routine assessment of mood in patients of BPD was found to provide similar results to traditional clinician interview-based assessments, suggesting that remote monitoring, even if not self-reported, can be a reliable alternative to clinic-based monitoring [65]. In 2011, the MONitoring, treAtment and pRediCtion of bipolAr disorder episodes (MONARCA) trial, a randomised controlled single-blind trial, was conducted to ascertain the efficacy of their custom-designed smartphone application in patient self-monitoring of BPD symptoms, in order to assess illness progression and identify early affective symptoms. The application was designed specifically for the assessment of subjective (mood, irritability, sleep) and objective (speech, social activity, physical activity, alcohol) changes in BPD as well as treatment adherence, providing a feedback loop between patients and providers. These combinations of subjective and objective symptoms were run through scoring frameworks to provide an objective means of assessing risk of deterioration, which was assessed by independent clinicians. Users were also assessed on universal and personalised early warning signs, such as sudden changes in behaviour [11]. Current research on the MONARCA system indicates that patients find it useful and easy to operate, indicating its feasibility as a self-monitoring tool [11]. A recent trial of 17 BPD patients using the MONARCA application for three months found that self-reported mood scores on the application correlated significantly with scores on the Hamilton Depression Rating Scale-17 (HDRS 17); for every 10-point increase on the HDRS-17, there was a 0.51-point decrease in the self-rated mood on the mood scale from 0 to −3 (*p* < 0.0001). There was a similar correlation between the HDRS-17 with the number of times a patient changed their cell tower ID, with a 10-point increase in HDRS-17 corresponding to a 4.8-times decrease in patients changing between cell towers (*p* = 0.020). This indicates that both subjective and objective measures in MONARCA correlate well with the HDRS-17 used by clinicians in monitoring BPD. However, there was no correlation found with the Young Mania Rating Scale (YMRS), the other mood scoring system used in the study [11].

Similar to MONARCA, the Self-monitoring and psychoeducation in bipolar patients with a smart-phone application (SIMPLe) project was designed to validate a custom smartphone application to [14]:

- Monitor symptoms and signs of BPD
- Empower self-management of BPD
- Offer customized psychoeducational content
- Identify early symptoms to prevent relapses and hospitalizations.

The study consisted of three phases: a feasibility study, a qualitative study, and a randomised-control trial to evaluate management of BPD with the SIMPLe application added to conventional management, as compared to conventional management alone. The application starts with five daily questions on mood, energy, sleep time, irritability, and medication adherence. It then compares the answers on a weekly basis to look for relevant mood changes. Should any be detected, the user is then directed to a more thorough questionnaire based on DSM-V criteria for mood disorders. The user is then given tailored psychoeducational advice. The user is also assessed on substance use and suicidal ideation. Should the latter prove to be a risk, an immediate notification is sent to the mental health team, and a call to emergency services is suggested by the application interface. A web-based interface is being developed to allow real-time monitoring of patients by the mental health team [14]. 

Another smartphone application, Social Information Monitoring for Patients with Bipolar Affective Disorder (SIMBA), has been produced [12]. In a similar fashion to MONARCA, this application aims to combine subjective patient-reported mood symptoms with objective measures of physical activity, based on GPS-detected movement, cell tower changes and accelerometer readings, and social activity, to monitor patients on BPD. The hypothesis was that depressive symptoms could be predicted by subjectively recorded low mood and low physical/social activity, while manic symptoms could be predicted conversely by recorded high mood and high physical/social activity. Thirteen patients were followed up for one year using the application. The results of the trial were varied, with specific aspects of the application showing correlation with depression or mania respectively. High reported mood on SIMBA correlated negatively with depressive symptoms as assessed by the HDRS (*p* < 0.001), but there was no correlation between the reported mood on the application and manic symptoms, indicating that users reporting high mood were less likely to be depressed, but their likelihood of mania could not be predicted. In terms of objective data tracked by SIMBA, distance travelled based on GPS measurements correlated negatively to mania assessed by the YMRS (*p* < 0.001), indicating that users with increased physical activity were less likely to exhibit manic symptoms. However, cell tower movements and device activity were not correlated with clinical symptoms. In terms of social activity, the number of calls made correlated positively to manic symptoms (YMRS) (*p* = 0.03), while the number of outgoing text messages sent correlated negatively with depressive symptoms (HDRS) (*p* < 0.001). This indicates that making more calls increased the likelihood of experiencing manic symptoms, while sending more text messages decreased the likelihood of experiencing depressive symptoms. It is unclear what the interplay between these two forms of communication is, and how they relate specifically to mood, but it is evident that social interaction and communication have a role to play in maintaining a healthy mood. The abovementioned correlations of objective measurements by the SIMBA application with mood symptoms remained significant when comparing within patients (as opposed to between patients) as well [12].

Along with MONARCA, SIMPLe, and SIMBA, more of such applications such as the “True Colours” application by the Oxford Group, are being trialled [22]. Facilitated Integrated Mood Management (FIMM) is a 5-session psychoeducational treatment aimed towards monitoring patients with BPD through mobile phone technology. Adult patients received 5 to 6 sessions of FIMM with pharmacotherapy. FIMM sessions focused on identifying early warning signs (EWS), maintaining regular daily and nightly routines, rehearsing mood management strategies, maintaining adherence to medications, and providing education about substance abuse. Altman Self-Rating Mania scale (ASRM) scores were seen to increase by a mean of 0.08 per week (95% CI: −0.05 to 0.21) whereas the Quick Inventory of Depressive Symptoms-Self Reported (QIDS-SR) scores had a downward trend of −0.11. These applications allow for patients to actively monitor themselves on a daily basis in both subjective and objective fashions. They also maintain feedback from the mobile phones to mental health teams, allowing for early intervention should there be any risk of severe disease relapse or suicidal ideation exhibited by the patients.

#### 5.1.2. Monitoring for Patients at Risk of BPD 

Mobile platforms, particularly smartphone applications, have been shown above to be useful in the regular monitoring of patients with BPD, overcoming one of the main difficulties in the clinical management of the disorder. Another major obstacle in management, as mentioned above, is the difficulty in assessing the risk of BPD in patients that present with mood symptoms, leading to late or incorrect diagnosis, which is known to reduce efficacy of treatment [30,31,32,33].

The Mobile Mood Diary (MMD) is an application developed for the assessment and monitoring of adolescents presenting with mood symptoms [13]. The application allows users to record their mood, energy levels, and sleep patterns, as well as make freeform text entries. This data can then be uploaded, at the user’s request, to an online interface which then allows them to track and visualise their recordings in graphical displays. The online interface also allows for personal text message reminders to be configured for individual users. While there is no objective data on the efficacy of this application in predicting the risk of mood disorders in these adolescent subjects, individual case reports indicate that both the patients and their clinicians developed a better understanding of their own mood symptoms, including personal triggers for worsening mood, which therefore led to better adherence to both the application as well as clinic appointments [13]. As mentioned above, one of the impediments to early diagnosis of BPD is the fact that patients have poor understanding of their symptoms and therefore seek clinical help too late [31]. Based on this, it can be argued that this application has the potential to help in early diagnosis of mood disorders in adolescents with impending bipolar affective disorder (BPAD). 

Currently, there is a paucity of literature on the use of mobile platforms in predicting and diagnosing BPD. This is likely to be because most research on the usefulness of mobile applications in BPD is currently being done on diagnosed patients, and is still ongoing. Regardless, the potential of these platforms in the assessment of risk of BPD is evident. The advantages offered by applications on the market for BPD patients, such as daily symptom monitoring, tracking of activity, personalised psychoeducation, as well as direct feedback to mental health professionals, is useful in undiagnosed BPD. It is therefore possible that such applications for the assessment and diagnosis of BPD will become more prevalent in the near future, helping to overcome the difficulty of early diagnosis seen in clinical practice. As it stands, however, current mobile applications are clearly designed for the monitoring of known BPD patients rather than monitoring those at risk of BPD, and more research into the role of mobile platforms in assessing at-risk patients, versus already diagnosed patients, is necessary.

### 5.2. New Markers of BPD for Effective Monitoring

As mentioned above, the traditional clinic-based assessments of patients with BPD is mostly based on retrospective accounts by patients about their mood and activity levels. Clinicians are unable to assess these patients objectively and in real-time, and these assessments are usually done in the form of observations made in the clinic setting as well as questionnaires [42,43]. However, with mobile platforms now able to deliver real-time data to mental health professionals, the objective assessment of BPD can be a possibility, if patient compliance permits. Physical and social activity levels now have direct correlates that can be tracked by mobile phone applications, and these essentially act as novel markers of disease progression. These can then be combined with subjective markers of mood into algorithms that can assess the risk of deterioration in these patients.

Many of the abovementioned smartphone applications that have been trialled on BPD patients are able to track such data. Both the MONARCA and SIMBA applications track specific behaviours and activities in their users as markers of physical and social activity per day, as illustrated in Table 3 [66]:

Through the use of built-in sensors in the smartphones, these applications are able to provide new markers of BPD progression in real-time that clinicians were previously unable to measure. Other applications have been designed to collect physiological sensors using wearable technologies. The personalised monitoring systems for care in mental health (PSYCHE) European project developed a sensorised T-shirt that records changes in physiological markers of the autonomic nervous system (ANS) [67]. The T-shirt can acquire the inter-beat interval time series, heart rate, and respiratory dynamics for long-term monitoring through the day and during sleep overnight. It works on the hypothesis that mood changes in patients with mood disorders correlate to changes in the ANS, and therefore that changes in biomarkers of the ANS can be used to assess changes in mood. While it is possible to make such measurements of heart rate and respiratory dynamics in clinic settings, these only allow for routine measurements at intervals rather than continuous real-time measurements, as offered by this technology. Three patients with BPD were followed for a period of ninety days, during which up to six monitoring sessions and psycho-physical evaluations were performed for each of them. The PSYCHE platform was able to give an accurate description of each individual’s mood states at given times, reinforcing the idea that these physiological markers of ANS change can be used in combination as novel markers of mood change, rather than as discrete measurements [67]. Other similar trials have been conducted using smartphone-based sensor technology. A trial of ten patients over ten weeks found that by using four different sensor-modalities on smartphones, the phones were able to detect changes in physiology corresponding to depressive and manic states, achieving recognition accuracies of 76% and state-change detection precision and recall of more than 97% [68]. More recently, smartphone applications have been developed to assess the relationship of sleep duration with mood symptoms in patients with BPD. Forty-one patients provided daily reports of sleep duration and affect collected via ecological momentary assessment with smartphones over eleven weeks. The trial found that increased cumulative day-to-day sleep duration variability was associated with increased severity in mood symptoms, reduced medication adherence, and greater negative affect [69]. In another trial, smartphones were used to collect voice features, automatically generated objective data on behavioural activities, and electronic self-monitored data from 28 patients with BPD [70]. 

Thus, either by allowing the recording of new types of objective data or by allowing for the combination of known physiological markers into new markers of mood states, mobile platforms have enabled patients and clinicians to monitor and assess their condition in more objective fashions.

### 5.3. Remote Management and Early Intervention

From above, the biggest impediment to treating BPD is late or misdiagnosis, resulting in delayed treatment [30,31,32,33]. Therefore, by potentially helping in earlier and correct diagnoses, mobile platforms could have a significant positive impact on treatment of BPD. There is room for digital platforms to incorporate aspects of treatment into their interfaces. Current evidence shows that psychological interventions and psychoeducation are very effective as adjuncts to pharmacological therapies, reducing the risk of relapses, improving medication adherence, and improving outcomes in patients [22,71,72,73]. In light of this, The Future Internet Social and Technological Alignment Research (FI-STAR) group has designed the Bipolar Patient Treatment Management (BPTM) platform, an online psychoeducation tool to help in the treatment of patients with BPD. The psychoeducation will be conducted via learning sessions (with assessment questionnaires) and psychotherapy exercises, with patients in the trial undergoing nine sessions, once a week. Through the online interface, patients will be able to communicate with mental health professionals and view multimedia content tailored to their performance in the exercises. While the results of the trial on this platform are not yet available, it is clear that it has the potential to allow for not just remote monitoring of patients with BPD, but also to provide active non-pharmacological treatment. As patients on the system are monitored daily and have channels through which communication with mental health professionals is always possible, it is likely that this platform will greatly assist in more timely interventions for patients at risk of deterioration [21].

Similar to this online platform is the abovementioned SIMPLe mobile application, which also produces detailed psychoeducational advice for users tailored to their specific mood states, as assessed by the application [14]. As with psychoeducation, cognitive behavioural therapy (CBT) has been shown to be an effective adjunct to pharmacological management, particularly in delaying or preventing relapses [74,75,76,77]. Previous research has demonstrated the usefulness of CBT delivered via digital platforms in addressing the psychological needs of bariatric surgery patients [69,70,71,72]. The team behind the development of personal digital assistants-based ecological momentary interventions to improve treatment adherence in BPD patients is now developing a smartphone version that will incorporate psychoeducation and CBT [64,78]. Interpersonal and social rhythm therapy (IPSRT) has been found to be effective in reducing the risk of recurrence in patients with BPD, as well as in improving occupational function in these patients [79,80]. This has led to the development of a smartphone application, MoodRhythm, based on IPSRT and augmented with objective data from phone sensors. The application aims to stabilise daily routines in patients to prevent acute episodes [13]. As with most of these applications, there is no data available from trials as to whether they are truly effective in helping treat BPD. However, by providing access to forms of treatment (psychoeducation, CBT, IPSRT) that have been shown previously to be effective in treatment, they have the potential improve management of the disorder. By allowing for daily monitoring of patients, regular psychological interventions and timely intervention from mental health professionals when necessary, these applications could not only help improve adherence to pharmacological treatment, but may also directly help in preventing relapses and recurrences.

Preventing relapse is a key goal of most interventions for BPD. Interventions that teach people to recognize and respond to early warning signs (EWS) are recommended by clinical guidelines worldwide [3,4,5], but implementation in routine clinical practice is poor [23]. ERPOnline is an intervention that teaches people with BPD to recognise early warning signs (EWS) of relapse. Key features of ERPOnline include a detailed analysis of triggers of previous mood episodes, personalised plan on how to manage triggers, detailed analysis of EWS of high mood to develop a relapse signature for mania as well as for low mood for depression, and an individualised summary of strategies to manage stress and regulate mood. In a single-blind randomised-control trial (RCT), it was found that access to ERPOnline was associated with improved EWS monitoring and development of positive beliefs about mood swings. However, further research is needed to compare the effectiveness of ERPOnline to current treatment. Nevertheless, ERPOnline offers a cheap and accessible option for patients seeking support after initial management. Alternatively, it can be used for people at an earlier stage of treatment who need psychoeducation to understand their mood swings, side effects of medications, and usefulness of monitoring in place of more expensive face-to-face psychological therapy. 

Lastly, the use of these platforms is also associated with improved patient well-being. *Living with Bipolar* is an online interactive recovery-informed self-management intervention, broadly based on the principles of Cognitive Behavioural Therapy. The results of the *Living with Bipolar* trial demonstrated that patients with intervention had improved medication adherence as well as improved physical and psychological quality of life (QoL) compared to control at both 3-months and 6-months. These findings were also reflected in Online mindfulness-based intervention for late-stage bipolar disorder (ORBIT), which studied patients in late-stage BPD. There was a statistically significant improvement in QoL in the intervention group compared to control (*t*(15) = 2.88, 95% CI: 0.89–5.98, *p* = 0.011). *Beating Bipolar* is another internet-based psychoeducational treatment that showed to improve scores in the psychological subsection of the World Health Organization Quality of Life Instruments (WHOQOL-BREF) score (*p =* 0.05). In addition, they also improve scores on functioning, insight, and depressive and manic episodes, as shown in the Bipolar Interactive Psychoeducation (BIPED) study. While these interventions improve QoL in the short-term, further work is required to establish the long-term impact of these interventions on treatment adherence, self-management skills, and QoL. 

## 6. Possible Difficulties with Digital Platforms

While the potential for mobile platforms to improve the diagnosis, monitoring, and treatment of BPD is clear, the use of these platforms still has to overcome some difficulties.

### 6.1. Lack of Large Group Trials

Firstly, there is the issue of whether there is enough data from large group trials to justify the marketing of these mobile applications. From the examples above, it is clear that these applications are all still in the trial phase. Early research has only been carried out on very small sample sizes. MONARCA was trialled on only 78 patients between the ages of 18 and 60, SIMPLe on 30 patients over the age of 18, and SIMBA on 13 patients aged 18 and above [11,12,14,70]. While the preliminary data from these small patient groups is promising, it is uncertain if similar results will be replicated in larger populations. Furthermore, specific groups of patients, such as those outside the age ranges specified, those with severe depression or mania and those with comorbid mental illnesses such as schizophrenia and learning difficulties, were excluded from these trials. Considering that many patients with mental illness present with more than one, and that BPD affects a wide age range of patients, it is unclear if these applications can be useful for any more than just stable patients with a single diagnosis, which is the patient group at least risk. 

### 6.2. Evidence-Base

Secondly, a systematic review of mobile applications found that many applications on the market do not have enough evidence-base to justify being used for patients [64]. Of 19 applications developed to provide information on BPD, only four credited the sources of their information, and 12 were repurposed e-books with the same information, though unattributed. Applications were assessed on the comprehensiveness of their psychoeducation by determining how many of 11 key psychoeducation statements they covered. On average, applications covered four out of the eleven. Two statements (regarding the importance of treatment adherence and development of action plans) were not addressed by any. Five applications contained no BPD-specific psychoeducation line with best practice, while seven applications were the most comprehensive, covering seven statements. However, these were non-cited reproductions of the same content. Finally, the applications were assessed for their concordance with evidence-based practice. This was assessed by noting how many of thirteen key evidence-based statements, based on BPD treatment guidelines, were covered by these applications. On average, only two of the thirteen were covered. Only three applications covered more than three of the statements, and the one app that covered seven statements provided unreferenced information. However, there was one application, covering eight statements, which contained organised and concise information that was referenced to NHS Choices. Worryingly, four applications contained incorrect information, including incorrectly defining the different subsets of BPD. Two applications contained dangerously wrong information, with one suggesting taking “a shot of hard liquor [an] hour before bed” to assist with sleep during a manic episode. The other indicated that BPD can “sometimes ... transfer to another relative if they spend too much time with you and listen to your depressive life” [64]. It is concerning that applications such as these, providing information that is not evidenced-based at best and dangerous to patients at worst, are available to users, suggesting that stronger vetting policies are required by application providers before releasing them into the market. There have in fact been proposals for national healthcare organisations, such as the National Health Service in England, to provide users with their own store of applications, similar to what is done with iTunes and Google Play [81]. This would ensure that medical health applications are vetted by such organisations before they are released into the market. However, this would create a huge burden of cost for these organisations and hence may not be feasible [82]. Currently, there have been proposals to develop specific guidelines for applications relating to healthcare, specifically a systematic self-certification model for the peer review of applications [83]. Such a model would be able to assist healthcare professionals in determining if an application is safe and applicable to patients. There are current models existing for the evaluation of the information presented in medical applications, such as the Silberg scale, which has been used in assessing applications relating to obesity management and bariatric surgery [84,85,86]. New mobile application rating tools, such as the Mobile App Rating Scale (MARS) have also been developed specifically for healthcare applications [87]. More specifically for psychiatric applications, an application known as “WikiGuidelines” has been conceptualised, with the purpose of providing ease of access to evidence-based guidelines specifically for mental health disorders [88]. Regulatory bodies such as the Food and Drug Administration (FDA) have already developed regulatory guidelines for mobile applications [89]. There is therefore hope that the issue of poor evidence-base in mobile health applications will soon be overcome.

### 6.3. Confidentiality, Securityand Ethical Concerns

Thirdly, there is the issue of confidentiality and security of patients’ private health information. Mobile health applications, such as the one mentioned above, function by collecting real-time data continuously from patients, storing them in the mobile platform and also making them available to health professionals. In essence, these devices become electronic health records (EHR) for patients. A recent review of privacy and security in mobile health application found that clinicians and patients are adopting mobile technologies to record data faster than providers are able to guarantee security and privacy [90]. Users of these smartphone applications tend to be unaware of the privacy and security aspects of these devices. The review raises concerns that new healthcare applications do not have enough security measures in place to ensure the privacy and safety of patients’ health information [91]. Information being stored in mobile platforms as opposed to secure clinic and hospital networks may be easier to obtain illegally, whether through physical theft or hacking of the platform that stores the information. Furthermore, many of these applications provide very personal and individualised information to those who are analysing this data. Applications that track the users’ movements through GPS or cell tower ID changes are, in effect, allowing users’ movements to be tracked at all times. While most of the studies state that such data would be collected and analysed by healthcare providers, it is unclear in what capacity these professionals function. The clinic setting allows for the development of trust between doctors and patients, which is important in obtaining information from patients. These new applications require users to essentially trust professionals they may have never met. Furthermore, it is unclear to what capacity healthcare and non-healthcare staff are involved in the analysis of user data. It is difficult to believe that all the raw data analysis would be carried out by healthcare providers alone, seeing as they may not be trained for such work. The application providers themselves may have staff involved in this effect. Thus, there are ethical concerns with regards to patient information being made available to professionals not directly involved in their care. 

### 6.4. Feasibility

As mentioned above, the use of mobile phone applications requires patients to regularly provide subjective data on their mood and consent to the recording of objective data, through the applications to healthcare professionals, who then have to analyse the data and provide the necessary information back to patients as first-line intervention. Compared to visiting clinicians once every few weeks for outpatient appointments, regular submission of information through mobile phones can turn into a very time-consuming activity. As mentioned above, trials of applications for BPD have only been carried out on very small sample populations. Though completion rates have been promising, it is unclear if these can be replicated with large groups of users. Users may find the process tedious and hence may simply stop using these applications. This is especially a problem in patients with moderate or severe disease. Thus, it is unclear how feasible these applications would be in larger patient populations in the long-term.

A considerable number of with BPAD patients have limited insight into their disorder, especially during periods of mania, which could affect compliance with the applications. Hence, it is important to highlight that these mobile applications are not meant to completely substitute in-patient and community psychiatric care, but to facilitate prompt recognition and easy monitoring. 

## 7. Conclusions

Table 4 summarises the key findings of this review:

In general, there is evidence to suggest that digital platforms, particularly mobile phone applications, can revolutionise both the monitoring and assessment of BPD. By allowing for remote monitoring and management, these platforms would be able to assist clinicians in managing patients beyond simply meeting them during routine clinical appointments, thereby augmenting current management protocols. However, care must be taken to ensure the privacy of patient information, and there is a need for large-scale randomised-control trials on more diverse patient populations, such as those with comorbid mental illnesses, to determine if these applications are truly useful for the majority of the patient population with BPD.

## Figures and Tables

**Table 1:** Studies on digital platforms used in the assessment, monitoring and management of BPD.

Study	Design	Sample Size	Sample Characteristics	Main Findings
MONARCA [11]	Prospective observational study	17	67.1% (*n* = 45) females 18–60 years (Median age 29.83 years)	Subjective and objective measures in MONARCA correlate well with the HDRS-17 used by clinicians in monitoring BPD
SIMBA [12]	Prospective observational study	13	61.5% (*n* = 8) females Mean age 47.2 years	High self-reported mood correlates negatively with depressive symptoms Distance travelled correlates negatively with mania No. of calls made correlates positively with manic symptoms No. of text messages sent correlates negatively with depressive symptoms
Mobile Mood Diary [13]	Observational study	9	55% (*n* = 11) females Mean age 13.6 years	Improved adherence to medications
SIMPLe [14]	RCT	Ongoing	18–65 years (Mean age)	Ongoing
Moodswings.net.au [15]	RCT	156	73% (*n* = 97) females Median age 39.87 years	Reduction in mood symptoms, improved functioning and improved QoL and medication adherence in intervention group
ORBIT [16]	RCT	26	75% females Median age 46.6 years	Statistically significant improvement in QoL in intervention group t(15) = 2.88, 95% CI: 0.89–5.98, *p* = 0.011
ERPOnline [17]	RCT	96	61.4% females Median age 43 years	ERPonline increased the frequency of monitoring early signs of mood change. Improved medication adherence at 12 and 48 weeks.
Living with Bipolar [18]	RCT	122	72% (*n* = 88) females 18–65 years (Mean age 43 years)	Improvement in psychological and physical domains of QoL, well-being in the intervention group compared to control
Beating Bipolar [19]	RCT	50	38% (*n* = 19) males Mean age 43 years	Improvement within the psychological subsection of the WHOQOL-BREF for the intervention group relative to the control group
BIPED [20]	RCT	100	Ongoing	Ongoing
FiSTAR [21]	RCT	25	Ongoing	Ongoing
FIMM [22]	RCT	19	68.4% (*n* = 13) females Mean age 37.2 years	Patients reported stable moods on the QIDS and ASRM over a 120-day period, and on average responded to 81% of the daily message prompts and 88% of the weekly QIDS and ASRM prompts.

MONARCA, MONitoring, treAtment and pRediCtion of bipolAr Disorder Episodes; ERP, Enhanced Relapse Prevention for Bipolar disorder; RCT, randomised controlled trial; WHOQOL-BREF, Brief World Health Organization Quality of Life; BIPED, The Bipolar Interactive Psychoeducation; FI-STAR, The Future Internet Social and Technological Alignment Research; FIMM, Facilitated Integrated Mood Management.

**Table 2:** Diagnostic and Statistical Manual of Mental Disorders, Fifth Edition (DSM-V) sub-classifications of bipolar disorder (BPD).

	Bipolar I	Bipolar II	Cyclothymia	Bipolar NOS
Depressive episodes	?	√	√√	?
Manic episodes	√	?	√√	?
Hypomanic episodes	?	√	√√	?
Others				Any disorder with BPD features not meeting criteria for others

Key: “√”—At least 1; “?”—Possibly at least 1; “√√”—Numerous.

**Table 3:** Markers tracked by MONARCA & SIMBA.

Application	MONARCA	SIMBA
Social activity	No. of outgoing/incoming calls/text messages	No. and duration of outgoing calls
	Speech duration during calls	No. of outgoing text messages
Physical activity	Accelerometer readings	Distance travelled (Global Positioning Satellite (GPS)-tracked)
		Cell tower movement
		Accelerometer readings

**Table 4:** Findings of this review on digital platforms for BPD.

Difficulties in Current Monitoring and Assessment of BPD	Potential Benefits of Mobile Platforms	Potential Difficulties with Mobile Platforms
Late and incorrect diagnoses	Continuous real-time monitoring	Only trialled on small, non-representative patient groups
Lack of objective data	Novel phenotypic markers which provide objective data	Poor evidence-base
Patients not seeking medical intervention	Remote psychoeducation and CBT	Lack of proper security to ensure confidentiality of patient information
	Potential for earlier diagnosis in at-risk patient groups	
